# New Strategies and Methods to Study Interactions between Tobacco Mosaic Virus Coat Protein and Its Inhibitors

**DOI:** 10.3390/ijms17030252

**Published:** 2016-02-26

**Authors:** Xiangyang Li, Zhuo Chen, Linhong Jin, Deyu Hu, Song Yang

**Affiliations:** State Key Laboratory Breeding Base of Green Pesticide and Agricultural Bioengineering, Key Laboratory of Green Pesticide and Agricultural Bioengineering, Ministry of Education, Guizhou University, Guiyang 550025, China; gychenzhuoy@sina.com (Z.C.); fcc.jinlh@gzu.edu.cn (L.J.); fcc.dyhu@gzu.edu.cn (D.H.); fcc.syang@gzu.edu.cn (S.Y.)

**Keywords:** coat protein, anti-TMV compounds, strategies and methods, interactions

## Abstract

Studies of the targets of anti-viral compounds are hot topics in the field of pesticide research. Various efficient anti-TMV (Tobacco Mosaic Virus) compounds, such as Ningnanmycin (NNM), Antofine (ATF), Dufulin (DFL) and Bingqingxiao (BQX) are available. However, the mechanisms of the action of these compounds on targets remain unclear. To further study the mechanism of the action of the anti-TMV inhibitors, the TMV coat protein (TMV CP) was expressed and self-assembled into four-layer aggregate disks *in vitro*, which could be reassembled into infectious virus particles with TMV RNA. The interactions between the anti-TMV compounds and the TMV CP disk were analyzed by size exclusion chromatography, isothermal titration calorimetry and native-polyacrylamide gel electrophoresis methods. The results revealed that assembly of the four-layer aggregate disk was inhibited by NNM; it changed the four-layer aggregate disk into trimers, and affected the regular assembly of TMV CP and TMV RNA. The four-layer aggregate disk of TMV CP was little inhibited by ATF, DFL and BQX. Our results provide original data, as well as new strategies and methods, for research on the mechanism of action of anti-viral drugs.

## 1. Introduction

Tobacco mosaic virus (TMV) is responsible for devastating diseases in major agricultural crops, including vegetables and tobacco, which often lead to high frequency of occurrence together with serious damage and enormous economic loss [1,2]. Based on these important and difficult challenges, researchers have exerted intensive efforts in discovering novel anti-TMV lead structures for plants, optimizing lead compounds to obtain commercially registered anti-TMV agent products and investigating new molecular targets. The abovementioned agents are divided into three types. The first type includesinactivating agents, which can destroy the morphology of virus particles, such as Antofine (ATF) [3,4]. The second type includes curative agents, which can inhibit virus replication and proliferation, such as Ningnanmycin (NNM) [5,6]. The third type includes immune and protective agents, which can induce resistance to plant diseases, such as Dufulin (DFL) [7] and Bingqingxiao (BQX) [8]. NNM is a microbial pesticide with active ingredients including cytosine nucleoside compounds isolated from *Streptomyces nourseivar*. xichangensis. Some reports have indicated that NNM is a good anti-viral drug [3], Han *et al.* reported that NNM inhibited the polymerization of TMV CP *in vitro*, in addition to multiple other activities affecting expression of various host genes that may contribute to systemic resistance to TMV in tobacco [9]. ATF is extracted from *Strobilanthe cusia*, and has strong interactions with TMV initial RNA, leading to assembly blocking of virus particles [4]. DFL is a plant anti-viral agent with a high activity against TMV and an immune activator in plants [7]. BQX has preventive and curative effects, which could change the profile of protein expression after TMV infection [8,9]. 

TMV CP include disk and helix formations [10]. The disk forms appeared in sodium phosphate buffer without TMV RNA; the helix forms appeared sodium phosphate buffer with TMV RNA. The TMV CP disk forms play an important role in forming TMV particles with both the TMV CP helix forms and TMV RNA [10]. Thus, TMV CP disk forms are potential targets of anti-viral compounds.

To further study the mechanisms of action of anti-TMV inhibitors, we obtained the four-layer aggregate disk forms of TMV CP as a target of the anti-TMV drugs *in vitro*. The four-layer aggregate disk of TMV CP can form rods, and can assemble into infectious virus particles with TMV RNA in 10 mM sodium phosphate and 100 mM sodium chloride at pH 7.2. After adding the anti-TMV drugs, the TMV CP disk could be disassembled into trimers by NNM, and could be disassembled into dimers by ATF, but not by BQX and DFL. After analysis using size exclusion chromatography (SEC) and native-polyacrylamide gel electrophoresis (native-PAGE) between NNM and the TMV CP disk, we found that NNM inhibited the assembly of TMV CP; it changed the disk of TMV CP into trimers. The isothermal titration calorimetry (ITC) results revealed that the hydrogen-bonding networks of TMV CP disk had interactions with NNM.

## 2. Results and Discussion

### 2.1. Formation of TMV CP Disk

#### 2.1.1. TMV CP Disk Formation Confirmed by SEC and Native-PAGE

**Figure 1:**
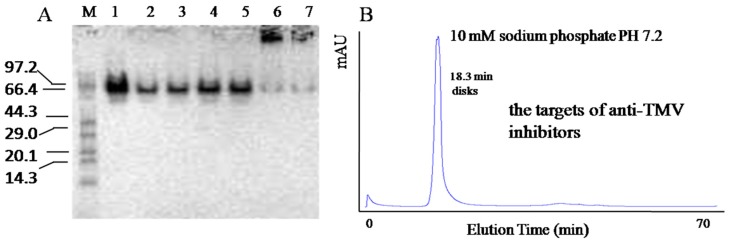
TMV CP disk (~34 subunits, ~595 kDa) were confirmed by native-PAGE and SEC. (**A**) M, marker proteins, listed as 97.2, 66.4, 44.3, 29.0, 20.1 and 14.3 kDa, lanes 1–7, purified TMV CP at 12 mg/mL (lane 1) or 6.8 mg/mL (lanes 2–7) was incubated in 10 mM sodium phosphate (pH 7.2) at 295 K for 0.5 h (lanes 1–3), 5 h (lanes 4, 5), or 24 h (lanes 6, 7); (**B**) 0.5 mM (6.8 mg/mL) TMV CP disk (~34 subunits, ~595 kDa) were confirmed by SEC, the peak time of TMV CP disk is 18.3 min.

To obtain TMV CP disk formation, the TMV CP gene was subcloned in a prokaryotic expression system. The TMV CP fused to a 6-His-tag produced in a prokaryotic expression system was purified using the 6-His-tag, which was then cleaved from the TMV CP using thrombin. The fresh TMV CP protein was observed primarily as tetramers (~4 subunits, ~70 KDa) using SEC in 10 mM sodium phosphate and 100 mM sodium chloride solution (pH 7.2).The disk (~34 subunits, ~595 KDa) was formed when the tetramers were incubated in 10 mM sodium phosphate and 100 mM sodium chloride solution (pH 7.2) at 295 K for more than 12 h (Figure 1A). TMV CP tetramers were incubated at 295 K for 24 h, and TMV CP disk were detected by native-PAGE. The TMV CP disk were confirmed by SEC (Figure 1B). This process of obtaining TMV CP disk is easier than traditional methods [11,12,13]. 

#### 2.1.2. Reconstructed TMV Observed by Transmission Electron Microscopy (TEM)

**Figure 2:**
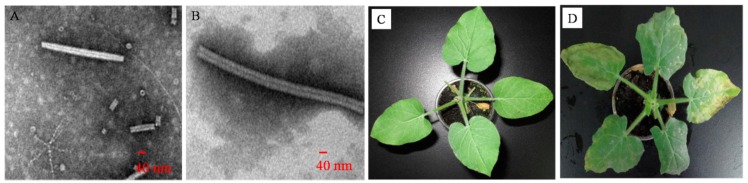
TMV CP disk and rod and reconstituted TMV virus particles were confirmed by TEM: (**A**) TMV CP (6.8 mg/mL) incubated in 10 mM sodium phosphate and 100 mM sodium chloride solution (pH 7.2) at 295 K for 24 h; (**B**) TMV CP (6.8 mg/mL) and TMV RNA (2 mg/mL) incubated in 10 mM sodium phosphate and 100 mM sodium chloride solution (pH 7.2) at 295 K for 24 h; (**C**) TMV CP disk and rod could not infect *Nicotiana glutinosa* (*N. glutinosa*) by mechanical inoculation; (**D**) the reconstituted virions could infect *N. glutinosa* by mechanical inoculation.

As described in the Experimental Section, the activation and function of TMV CP were confirmed using TEM. TMV CP refolding and further self-assembly was carried out at a protein concentration of 6.8 mg/mL. When TMV CP protein concentration was 6.8 mg/mL at 295 K for 24 h, disk and rod were observed by TEM (Figure 2A), and reconstituted TMV was obtained in the solutions when 2 mg/mL TMV RNA was added and incubated at 295 K for 24 h (Figure 2B). The freshly purified TMV CP oligomers self-assembled into TMV CP disk in the appropriate solutions for reconstruction into newly infective viruses (Figure 2C,D). Therefore, the freshly purified TMV CP may be regarded as the target of anti-TMV compounds.

### 2.2. Interactions of Anti-TMV Drugs and TMV CP Disk

#### 2.2.1. Interactions between Anti-TMV Drugs and TMV CP Disks Using ITC

**Figure 3:**
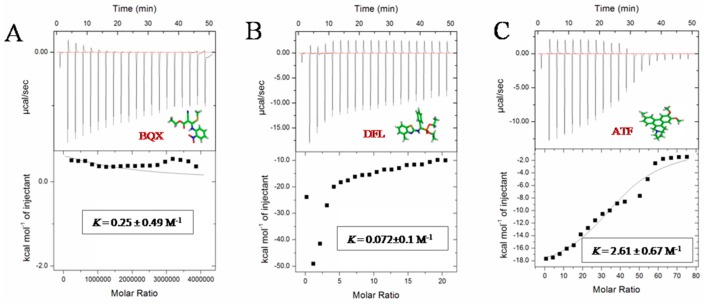

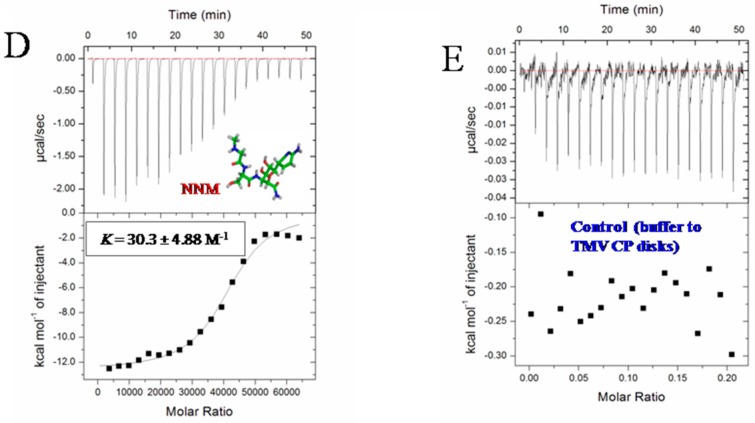
ITC profiles for the titration of TMV CP with (**A**) BQX; (**B**) DFL; (**C**) ATF; (**D**) NNM and (**E**) buffer (10 mM sodium phosphate, pH 7.2) with the TMV CP disks the top panels show the raw heat flow-to-time data; the bottom panels show the calculated molar heats of ligand binding.

ITC experiments were performed under the following conditions: 10 mM sodium phosphate and 100 mM sodium chloride at pH 7.2 to explore the energetic association of the compounds BQX, DFL, ATF and NNM with the TMV CP disk. The raw data of the heat change over time (top) and the plots of the integrated, corrected molar heats *versus* the ligand-to-protein ratios (bottom) are shown in Figure 3. The results showed that NNM and ATF had a micromole affinity for the TMV CP disk: Analysis by ITC revealed that one TMV CP disk interacted with 4100 to 4632 NNM molecules, and NNM bound to TMV CP disk with a dissociation constant (*K*_d_) of 3.3 μM (Figure 5D). The titration data indicated an apparent negative enthalpy value (△G ≈ −7.5) when NNM bound to TMV CP disk (Table 3), which indicated that the NNM-TMV CP disk complex was stable; one TMV CP disk interacted with 39 to 40 ATF molecules, and ATF bound to TMV CP disk with a dissociation constant (*K*_d_) of 38.8 μM (Figure 5C). The titration data also indicated an apparent negative enthalpy value (△G ≈ −5.6) when ATF bound to TMV CP disk, which indicated that the ATF-TMV CP disk complex was stable, but less sensitive than NNM. However, the affinities between other anti-TMV drugs (BXQ and DFL) and TMV CP disk were less sensitive (Figure 5A,B) than NNM and ATF and were within the 400–13,900 μM range.

#### 2.2.2. Interactions between Anti-TMV Drugs and TMV CP Studied by Native-PAGE

**Figure 4:**
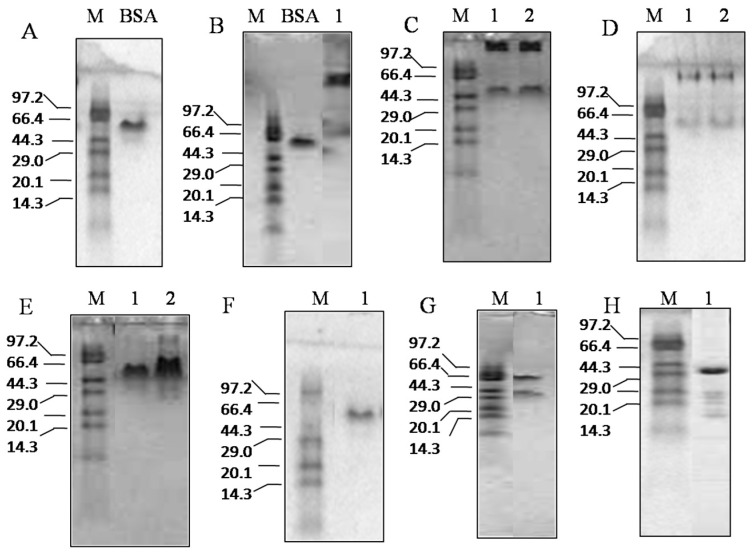
Interactions between the TMV CP disk and the anti-TMV compounds by native-PAGE; all the mixtures with purified TMV CP and anti-TMV compounds was incubated in 10 mM sodium phosphate (pH 7.2) at 295 K for 30 min: (**A**) Protein markers (M) are listed as 97.2, 66.4, 44.3, 29.0, 20.1 and 14.3 kDa, BSA is used as a marker control (66 kDa); (**B**) BSA was used as a marker control (66 kDa), 0.5 mM (6.8 mg/mL) TMV CP disk were used as a protein control (~34 subunits, ~595 kDa); (**C**) Lane 1: 0.5 mM TMV CP disk were mixed with 5 mM DFL (containing 2.5% DMSO). Lane 2: 0.5 mM (6.8 mg/mL) TMV CP disk were mixed with 5 mM BQX (containing 2.5% DMSO); (**D**) Lane 1: 0.2 mM TMV CP disk were mixed with 2 mM DFL (containing 2.5% DMSO). Lane 2: 0.2 mM (2.7 mg/mL) TMV CP disk were mixed with 2 mM BQX (containing 2.5% DMSO); (**E**) Lane 1: 0.5 mM TMV CP disk were mixed with 5 mM NNM. Lane 2: 0.5 mM (6.8 mg/mL) TMV CP disk were mixed with 5 mM ATF; (**F**) 0.2 mM TMV CP disk were mixed with 2 mM NNM; and (**G**) 0.2 mM TMV CP disk were mixed with 2 mM ATF; (**H**) 0.2 mM TMV CP dimers were used as a protein control (35 kDa).

Native-PAGE was carried out in the presence of 0.5 mM TMV CP disk and 5 mM DFL containing 2.5% DMSO, BQX containing 2.5% DMSO, AFL and NNM separately. The results showed that DFL and BQX could not destroy the TMV CP disk, whereas NNM could change TMV CP disk into trimers and ATF could change the TMV CP disk into dimers (Figure 4).

#### 2.2.3. Interactions between Anti-TMV Drugs and TMV CP Studied by SEC

**Figure 5:**
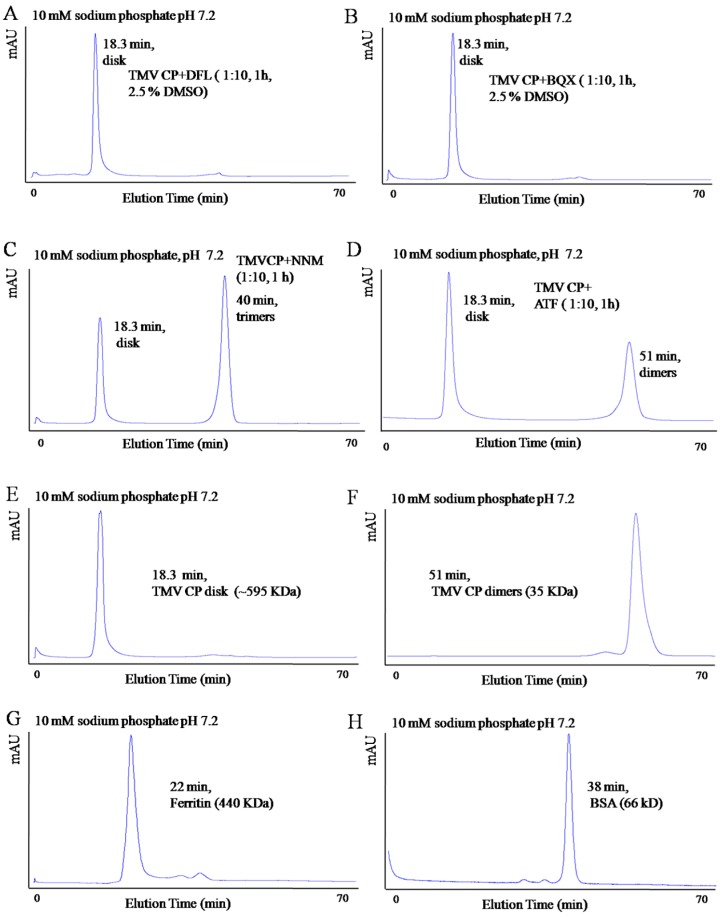
Interactions between the TMV CP disk and the anti-TMV drugs, as analyzed by SEC: (**A**) 0.5 mM (6.8 mg/mL) TMV CP disk were mixed with 5 mM DFL (containing 2.5% DMSO), and then incubated in 10 mM sodium phosphate and 100 mM sodium chloride solution (pH 7.2) at 295 K for 1 h; (**B**) 0.5 mM (6.8 mg/mL) TMV CP disk were mixed with 5 mM BQX (containing 2.5% DMSO) and then incubated in 10 mM sodium phosphate and 100 mM sodium chloride solution (pH 7.2) at 295 K for 1 h; (**C**) 0.5 mM (6.8 mg/mL) TMV CP disk were mixed with 5 mM NNM and then incubated in 10 mM sodium phosphate and 100 mM sodium chloride solution (pH 7.2) at 295 K for 1 h; and (**D**) 0.5 mM (6.8 mg/mL) TMV CP disk were mixed with 5 mM ATF and then incubated in 10 mM sodium phosphate and 100 mM sodium chloride solution (pH 7.2) at 295 K for 1 h; (**E**) the peak time of TMV CP disk is 18.3 min; (**F**) the peak time of TMV CP dimers is 51 min; (**G**,**H**) are protein markers for size-exclusion chromatography, the peak time of ferritin is 22 min, and the peak time of BSA is 38 min.

In the SEC experiments, TMV CP disk were mixed with 5 mM DFL (containing 2.5% DMSO), 5 mM BQX (containing 2.5% DMSO), 5 mM NNM and 5 mM ATF separately and incubated in 10 mM sodium phosphate and 100 mM sodium chloride solution (pH 7.2) at 295 K for 1 h. The TMV CP disks were not disassembled into oligomers by DFL and 5 mM BQX (both containing 2.5% DMSO); however, TMV CP disk were disassembled into trimers by NNM and disassembled into dimers by ATF (Figure 5).

**Figure 6:**
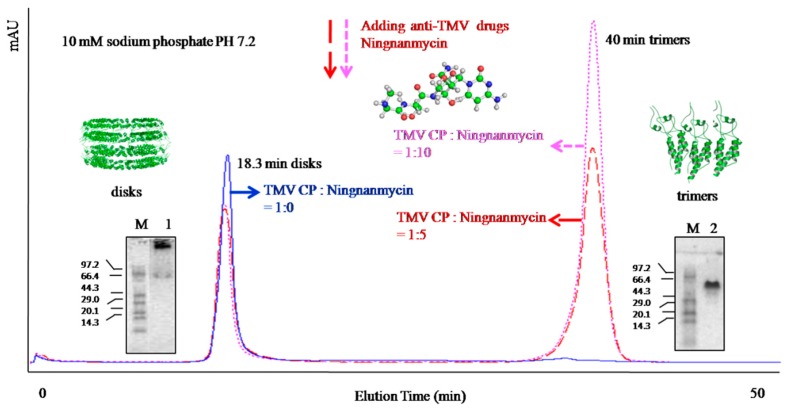
Prediction model between NNM and the TMV CP four-layer aggregate disk. Protein markers are listed as 97.2, 66.4, 44.3, 29.0, 20.1 and 14.3 kDa, lane 1 is TMV CP disk, and lane 2 is TMV CP trimers.

The concentrations of NNM solution were adjusted for further investigation of the interactions between TMV CP disk and NNM. When the ratio of TMV CP disk to NNM was 1:5, few TMV CP disks were disassembled into trimers; when the ratio was 1:10, most TMV CP disks were disassembled into trimers (Figure 6). The results imply that NNM could destroy the interlayer hydrogen-bonding networks in the four-layer aggregate of TMV CP disk.

### 2.3. In Vivo Assays of Anti-TMV Drugs and Reconstituted TMV Virus

Based on the mechanical inoculation methods of reconstituted TMV virus with anti-TMV drugs, NNM was verified to have a very good curative activity against TMV (60.6% in 500 μg/mL and 30.1% in 100 μg/mL) and ATF was verified that it has curative activity against TMV (61.1% in 500 μg/mL and 27.6% in 100 μg/mL), better than BQX and DFL.

## 3. Experimental Section

### 3.1. Preparation of Compound Samples

NNM was kindly supplied by Chen Jiaren of the Chengdu Biology of Chinese Academy of Sciences; ATF by Wang Qingmin of the Research Institute of Elemento-Organic Chemistry; DFL and BQX were designed and synthesized in our laboratory.

### 3.2. Obtaining TMV CP Disk

#### 3.2.1. Construction of Recombinant Plasmid Containing TMV CP 

TMV was purified using the method by Gooding [10] and modified by Shire [11,12]. TMV RNA was extracted from the purified virus by treatment with phenol (Solarbio, Shanghai, China) and sodium dodecyl sulfate (Solarbio, Shanghai, China) [14,15]. TMV RNA was reverse transcribed using primer 1 in 50 mmol/L Tris (Sangon, Shanghai, China) at pH 8.0, 8.0 mmol/L magnesium chloride (Sinopharm, Beijing, China), 75 mmol/L potassium chloride (Sinopharm, Beijing, China), 10 mmol/L dl-dithiothreitol (Sangon, Shanghai, China), 1.0 mmol/L dNTPs (Sangon, Shanghai, China), 0.5 unit/μL AMV reverse transcriptase (TaKaRa, Shiga, Japan), and 1.0 unit/μL RNase inhibitor (TaKaRa, Shiga, Japan) for 1.5 h at 315 K to generate the full-length viral cDNA sequence. Using the viral cDNA and primers 1 and 2 (Table 1), TMV CP sequences were amplified by PCR technology. The dsDNA of the correct length was purified and identified using 1% agarose gel electrophoresis (Sangon, Shanghai, China). Plasmid pET28a (Novagen, Darmstadt, Germany) and TMV CP cDNAwere digested with Nde I (NEB, Kallang, Singapore, 10 units/μL)/Xho I (NEB, Kallang, Singapore, 10 units/μL) and cloned into the same sites in pET28a. *Escherichia coli BL_21_(DE_3_)-plysS* (Novagen, Darmstadt, Germany) cultures were transformed into vectors involving the aforementioned recombinant plasmid.

#### 3.2.2. Expression and Purification of TMV CP

Expression plasmids were grown in Luria–Bertani medium containing 30 μg/mL kanamycin at 310 K until the OD_600_ reached 0.7. After cooling cultures to 289 K, the expression product was induced by addition of 0.5 mM Isopropyl *β*-d-1-thiogalactopyranoside (IPTG), and cultures were subsequently incubated for 16 h. The cells were harvested by centrifugation and resuspended in 40 mL of lysis buffer (100 mM sodium chloride (Sinopharm, Beijing, China) and 50 mM phosphate buffer (Sinopharm, Beijing, China) (pH 8.0) and 10 mM *β*-mercaptoethanol (Sinopharm, Beijing, China)). The cells were then thawed, lysed using a sonicator, and centrifuged at 12,000 rpm for 30 min at 277 K. The supernatant was passed through 0.22-mm syringe filters (Millipore, Darmstadt, Germany, UFC501096), loaded onto a Ni Sepharose high-performance column (GE Healthcare, Little Chalfont, Buckinghamshire, UK, 5 mL), washed with five column volumes of 40 mM imidazole (Sangon, Shanghai, China), and eluted with 400 mM imidazole (Sangon, Shanghai, China). The flow-through was concentrated in an AmiconUltra centrifugal filter device (Millipore, Darmstadt, Germany, UFC501096) with a 10-kDa filter, and then loaded onto a HiLoad 16/60 Superdex 200 pg column equilibrated in the dialysis solution (10 mM sodium phosphate (Sinopharm, Beijing, China). and 100 mM sodium chloride solution (Sinopharm, Beijing, China), pH 7.2). After thrombin digestion of 6-His-tags at 277 K overnight, the TMV CP was further purified by SEC using a Superdex 200 column (GE Healthcare, Little Chalfont, Buckinghamshire, UK, 120 mL) in a buffer containing 10 mM sodium phosphate buffer and 100 mM sodium chloride solution at pH 7.2. The protein was then concentrated to 1 to 10 mg/mL for the biochemistry trials using Amicon Ultra centrifugal filter units (Millipore, Darmstadt, Germany, UFC501096) with a 10-kDa molecular weight cutoff. The target proteins were briefly stored at 277 K.

#### 3.2.3. Formation of TMV Disk

The purified proteins were incubated in 10 mM sodium phosphate and 100 mM sodium chloride solution (pH 7.2) at 295 K for 24 h to obtain the four-layer aggregate disk [16,17,18,19,20]. The disk forms were confirmed by SEC and native-PAGE. 

#### 3.2.4. Reconstituted Virus of TMV CP

One milliliter of purified TMV CP disk (6.8 mg/mL) solution (10 mM sodium phosphate and 100 mM sodium chloride solution, pH 7.2) was mixed with 0.2 mL of purified TMV RNA (2 mg/mL) and incubated at 295 K for 24 h. The suspensions were centrifuged at 5000 rpm for 1 min, and then the reconstituted virus was obtained [13,21,22].

### 3.3. TEM Imaging

TMV RNA and the self-assembled TMV CP disk were incubated as mentioned previously. 20 μL of the mixed solution was deposited onto a 300-mesh Formvar-carbon-coated copper grid for 2 min, followed by rinsing with ddH_2_O. The grid was then stained with 20 μL of 2% aqueous solution of tungstophosphoric acid (Sinopharm, Beijing, China) for 90 s as a negative stain [23,24,25,26,27,28]. Images were obtained at the Zunyi Medical University Electron Microscope Lab using a Hitachi H-7650 transmission electron microscope with 80 kV accelerating voltage.

### 3.4. Interactions of Anti-Viral Drugs and TMV CP

Given that TMV CP disks are regarded as the target of small-molecule compounds [6], we studied the interactions between TMV CP disk and anti-TMV compounds 0.5 mM of the small-molecule compounds NNM, ATF, DFL and BQX were respectively added to 0.5 mM (6.8 mg/mL) TMV CP disk (Table 2), and then incubated for 30 min. The solution interaction was then investigated by SEC, 17% native-PAGE, and ITC methods.

#### 3.4.1. SEC

SEC was performed at room temperature using a calibrated Superdex 200 10/300 GL column (GE Healthcare, Little Chalfont, Buckinghamshire, UK) attached to an AKTA purifier fast protein liquid chromatography system (GE Healthcare, Little Chalfont, Buckinghamshire, UK). The column was equilibrated with a solution containing 10 mM sodium phosphate and 100 mM sodium chloride at pH 7.2. The molecular mass standards (Bio-Rad, Hercules, CA, USA) used include thyroglobulin (669 kDa), ferritin (440 kDa), BSA (67 kDa), *β*-lactoglobulin (35 kDa), ribonuclease A (13.7 kDa), cytochrome (13.6 kDa), aprotinin (6.51 kDa), and vitamin B12 (1.36 kDa). Protein was monitored by absorbance at the wavelength of 280 nm.

#### 3.4.2. Native-PAGE

Native-PAGE was performed on ice with the TMV CP samples that were equilibrated overnight in a buffer containing 10 mM sodium phosphate and 100 mM sodium chloride at pH 7.2. 20 μL of the sample was treated with 20 μL of 2× loading buffer, including 12.5% 0.5 M Tris-HCl (*v*/*v*) at pH 6.8, 0.5% bromophenolblue (*w*/*v*), and 30% glycerin (*v*/*v*). Subsequently, 8 μL of the samples and 4 μL of the protein marker were loaded on a native-PAGE gel (4% stacking and 17% separating gel). Electrophoresis was run at 1× native-PAGE buffer (Tris-Gly, pH 8.8) at 273 K for 1 h [29]. After native-PAGE electrophoresis, the lane was stained with Coomassie blue [30,31] to locate the protein, and then destained with methanol and glacial acetic acid.

#### 3.4.3. ITC

ITC binding experiments were performed using an ITC 200 Micro Calorimeter at 291 K. All proteins were dialyzed against a buffer for 3 day prior to forming the TMV CP disk; the buffer contained 10 mM sodium phosphate, 100 mM sodium chloride at pH 7.2 or 10 mM sodium phosphate, 100 mM sodium chloride at pH 7.2, 2.5% DMSO. Aliquots of the anti-TMV compounds at 5 mM (syringe) were injected into TMV CP solutions at 0.5 mM (cell). Data were processed using Origin software, and binding isotherms were calculated based on a one-site binding model. A single titration was conducted for TMV CP. The *K*_d_ error values were based on the sum of square deviations between the nonlinear regression curve and the experimental data (Table 3) [32,33].

### 3.5. In Vivo Assays

We performed the efficacy of BQX, DFL, ATF and NNM on reconstituted TMV virus infection by *N. glutinosa* by mechanical inoculation. The leaves on *N. glutinosa* of the same ages were selected. The reconstituted TMV with 58.8 μg/mL concentration was dipped and inoculated on the whole leaves. Then the leaves were washed with water and dried. The compound solution (100 and 500 μg/mL) was smeared on one side and the buffer was smeared on the other side for control. The local lesion numbers were then recorded 3–4 days after inoculation. For each compound, three repetitions were conducted to ensure the reliability of the results. The *in vivo* assays were showed in (Table 4) as follows.

## 4. Conclusions

TMV CP disk were used as targets of anti-viral compounds. In our study, TMV CP disk were disassembled into trimers when the NNM solution concentrations increased; and TMV CP disk were partly disassembled into trimers or dimers when ATF was added. By contrast, the disk showed little disassembly into trimers or dimers with the addition of DFL and BQX. The results demonstrated that there were interactions between NNM and TMV CP disk, and that interactions between the inter-subunits and layers of the TMV CP disk were disrupted by NNM. We speculate NNM replaced the binding sites in the disk and disrupted the interactions between the inter-subunits and layers of the TMV CP disk.

To the best of our knowledge, this study is the first to clearly show the inhibition of the TMV assembly through a small-molecule-protein interaction. This approach provides original data, as well as alternative strategies and methods, which can be used in anti-viral drug mechanism research.

## Figures and Tables

**Table 1:** DNA sequences of the primers.

Primer Name	Primers
Primer 1	5’-GGAATTCCATATGTCTTACAGTATCACTACTCC-3’
Primer 2	5’- CCGCTCGAGTCAAGTTGCAGGACCAGAGG-3’

**Table 2:** Proportions of TMV CP disk and anti-TMV compounds.

Drug Name	Anti-Plant Viral Compound	Compound Concentration (mM)	Protein Concentration (mM)
DFL *^a^*	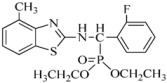	0.5	5
NNM *^a^*	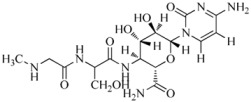	0.5	5
ATF *^a^*	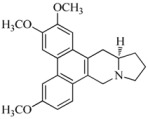	0.5	5
BQX *^a^*	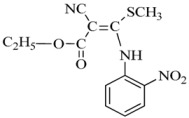	0.5	5

*^a^* Pure product.

**Table 3:** Thermodynamic parameters of the TMV CP and compounds interaction by ITC *^b^*.

Comp	N	*K*10^4^ M^−1^	*K*_d_ μM	ΔH kcal mol^−1^	TΔS kcal mol^−1^	ΔG kcal mol^−1^
BQX	1 ± 0	0.25 ± 0.49	400	5361 ± 9547	5364	−3
DFL	1 ± 0	0.072 ± 0.1	13,900	3400 ± 4240	4172	−772
ATF	39 ± 1	2.61 ± 0.67	38.8	−19.5 ± 0.12	−13.9	−5.6
NNM	4100 ± 532	30.3 ± 4.88	3.3	−12.6 ± 0.23	−5.1	−7.5

*^b^* The experiment was performed by titrating 10 mM compounds into 0.5 mM TMV CP disk. The ITC data were fitted to a one-set-of-sites model, errors from the fitting were shown.

**Table 4:** The results of preventative assay of the anti-TMV compounds.

Compounds	Inhibition Rate (%) *^c^*	Inhibition Rate (%)
	100 μg/mL	500 μg/mL
BQX	18.2 ± 2.4	47.7 ± 7.1
DFL	14.0 ± 4.8	51.0 ± 5.2
ATF	27.6 ± 6.7	61.1 ± 8.4
NNM	30.1 ± 7.8	60.6 ± 7.7
Buffer *^d^*	7.9 ± 1.3	10.5 ± 2.3

*^c^* The experiment was inoculated by means of half leaf, Inhibition rate (%) = (*av* local numbers of control (not treated with compounds) − *av* local numbers of treatment with compounds)/*av* local numbers of control (not treated with compounds); *^d^* In order to avoid errors, when we used means of half leaf, the treatment half and the opposing half were used alternatively, the buffer is 10 mM sodium phosphate and 100 mM sodium chloride solution (pH 7.2), errors were shown.
